# Professional autonomy among nurses in Saudi Arabian critical care units

**DOI:** 10.1186/s12912-023-01390-x

**Published:** 2023-06-29

**Authors:** Majed M. Alruwaili, Fuad H. Abuadas

**Affiliations:** 1 Nursing Administration & Education Department, College of Nursing, Jouf University, Sakaka, 72388 Saudi Arabia; 2 Community Health Nursing Department, College of Nursing, Jouf University, Sakaka, 72388 Saudi Arabia

**Keywords:** Professional autonomy, Patient care decisions, Unit operation decisions, Nurses, critical care units

## Abstract

**Background:**

Professional autonomy is essential in expanding the scope of nursing practice and has been recognized as a top nursing priority.

**Objective:**

This study aims to assess Saudi nurses’ autonomy level in critical care settings and examine the influence of sociodemographic and clinical characteristics on their autonomy level.

**Methods:**

A correlational design and a convenience sampling approach were used to recruit 212 staff nurses from five Saudi governmental hospitals in Jouf region of Saudi Arabia. The data were collected through a self-administered questionnaire composed of two sections, including sociodemographic characteristics and the Belgen autonomy scale. The Belgen autonomy scale used in this study measures nurses’ autonomy levels and consists of 42 items rated on an ordinal scale. The scale’s minimum score of 1 indicates nurses with no authority, while the maximum score of 5 indicates nurses with full authority.

**Results:**

Descriptive statistics revealed that nurses in the sample had a moderate overall work autonomy (M = 3.08), with higher autonomy in patient care decisions (M = 3.25) compared to unit operations decisions (M = 2.91). Nurses had the highest level of autonomy in tasks related to preventing patient falls (M = 3.84), preventing skin breakdown (M = 3.69), and promoting health activities (M = 3.62), while they had the lowest level of autonomy in ordering diagnostic tests (M = 2.27), determining the day of discharge (M = 2.61), and planning the unit’s annual budget (M = 2.22). The multiple linear regression model (R2 = 0.32, F (16, 195) = 5.87, p < .001) showed that education level and years of experience in critical care settings were significantly related to nurses’ work autonomy.

**Conclusion:**

Saudi nurses in acute care settings have moderate professional autonomy, with higher autonomy in making patient care decisions than unit operations decisions. Investing in nurses’ education and training could increase their professional autonomy, leading to improved patient care. Policymakers and nursing administrators can use the study’s results to develop strategies that promote nurses’ professional development and autonomy.

## Background

Professional autonomy is a multidimensional concept that has been contentiously confounded with other main concepts like independence, self-governance, and accountability [1–4]. Nowadays, professional autonomy is considered a main nursing professional priority in expanding the scope of the nursing practice [1–3]. Clinical and professional autonomy are the two main recognized categories of nurses’ professional autonomy [4]. Clinical autonomy is the capacity of staff nurses to operate beyond the realm of accepted practice and make decisions regarding patient treatment [4]. Meanwhile, professional autonomy is referred to as participation in developing and designing care processes to enhance nursing quality and patient safety [5]. While nurses have professional and clinical autonomy in their practice, there are also some boundaries to their autonomy [3, 6, 7]. For instance, nurses must practice within the legal and ethical context that governs their profession [3, 6, 7]. They must also follow the policies and procedures of the healthcare organization where they work [3, 6]. Additionally, they may need to consult with other health professionals when making certain decisions related to patient care [3, 7].

Nursing duties in healthcare settings are becoming more complicated and intertwined with many jobs, necessitating autonomy in their professional status [1, 5, 8, 9]. Professional autonomy assumes that nursing staff establish their own principles and act autonomously based on their own professional judgment, and it denotes that nurses can make their own professional decisions and have the right and responsibility to act in accordance with the nursing professional standards [1, 3, 9, 10]. Enhancing the autonomy of nurses is considered an essential factor that might improve the implementation of evidence-based practice [11] and can lead to positive outcomes for nurses (improve satisfaction), patients, and healthcare organizations [2, 12].

Autonomy is an important aspect of nurses’ professional practice, but the literature does not consistently support its direct relationship with turnover rates [5, 8, 13, 14]. While autonomy can contribute to job satisfaction and reduced turnover intentions, it is not necessarily the sole major factor influencing high nurse turnover rates [5, 13, 15]. Several factors have been identified as contributing to nurse turnover, such as workload, job-related stress, poor leadership, lack of support, limited opportunities for career advancement, and inadequate resources and staffing levels [8, 13, 14, 16, 17]. Some studies suggest that higher autonomy and decision-making authority levels can lead to job satisfaction and reduced turnover intentions [5, 8, 14, 17]. Nurses who have more control over their practice and are involved in decision-making processes may experience increased satisfaction and reduced turnover [8, 14]. However, the relationship between autonomy and turnover is complex, influenced by factors such as the alignment of desired and actual autonomy levels and variations across nursing specialties and settings [8, 13]. Developing competency in daily decision-making and gaining the courage to engage in advanced practice are two things that nurses must do, particularly those nurses who are working in acute care settings [1, 10, 11]. Therefore, Decision-making autonomy was recognized as an essential concept in some studies, particularly in acute care settings [1, 10, 15, 18, 19].

While professional autonomy is essential for all nurses, it is especially crucial for those working in acute care settings as they face complex and urgent situations requiring quick decision-making and critical thinking skills [1, 2, 5, 20]. Acute care nurses must navigate through the high-stress and fast-paced environment of acute care settings to provide quality patient care [1, 10, 11, 21, 22]. In contrast, nurses in wards often have more predictable routines and a lower degree of patient acuity [23]. Therefore, studying professional autonomy, specifically among acute care nurses, can provide a more detailed understanding of how autonomy impacts their daily work and can contribute to developing targeted interventions to enhance their professional autonomy [19, 24]. Considering the unique challenges and demands of acute care settings, studying professional autonomy among this population of nurses can provide valuable insights into the factors that affect their job satisfaction, retention, and overall well-being [8, 15, 25–31]. Ultimately, understanding the importance of professional autonomy among acute care nurses can inform strategies to improve patient outcomes, enhance the quality of nursing care, and promote a positive work environment for nurses in these critical settings [5, 8, 15, 20].

## Theoretical framework

**Fig. 1:**
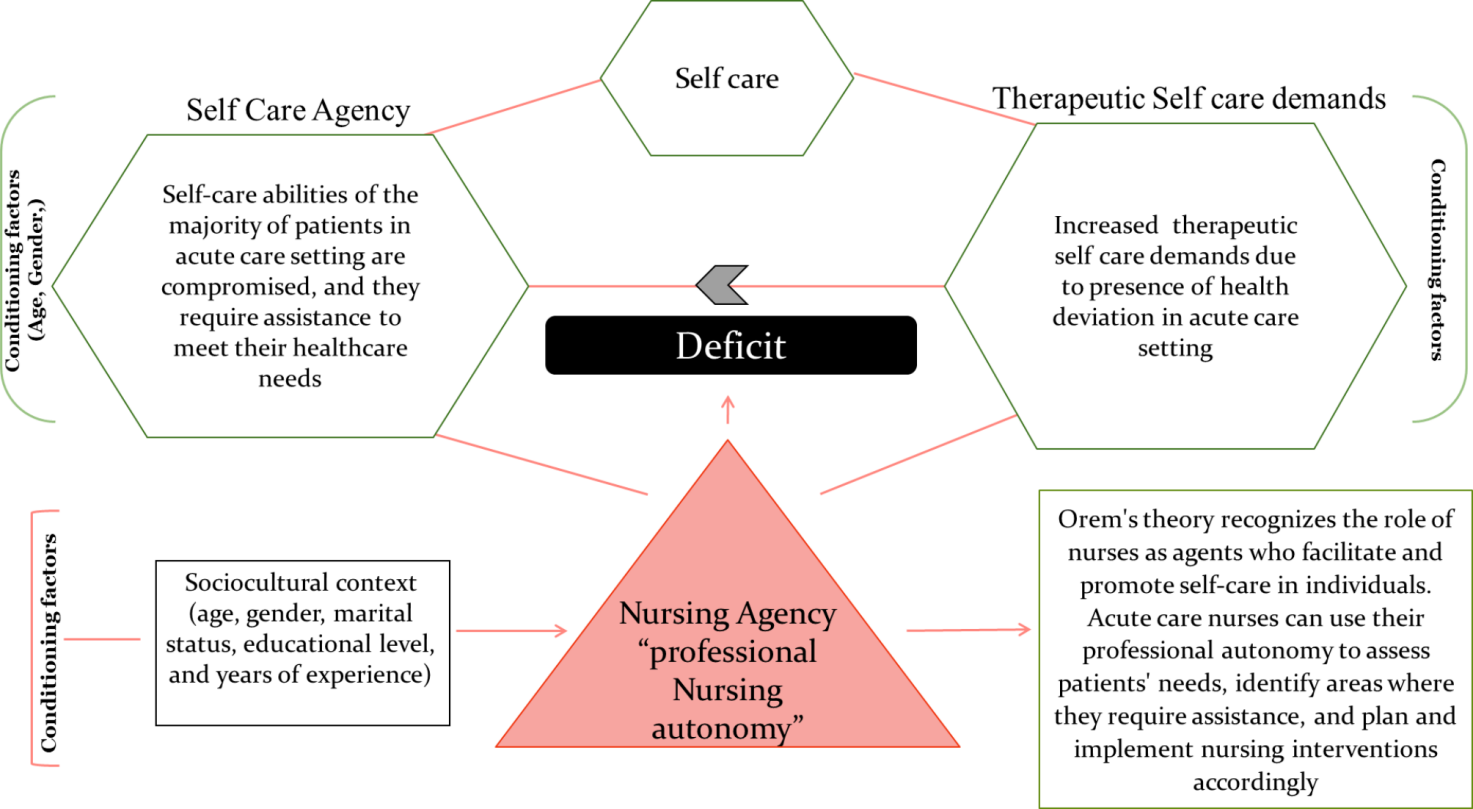
The relationship between the study variables and Orem’s general theory of nursing

Orem’s general theory of nursing [32–34] recognizes that individuals have varying degrees of self-care abilities, and when these abilities are compromised, nurses intervene to provide care [34]. In acute care settings, patients may experience limitations in their self-care abilities due to their health conditions or the acute nature of their illness [34]. Nurses play a crucial role in assessing these self-care deficits through comprehensive physical, psychological, and social evaluations [33]. By understanding the specific areas where patients require assistance, nurses can effectively tailor their interventions to address those deficits [33]. Orem emphasizes that nurses possess nursing agency, which refers to their ability to design and produce nursing care for patients [32–34]. Professional autonomy enables nurses to exercise their nursing agency by making informed judgments, implementing evidence-based knowledge and skills, and coordinating healthcare services [34]. In acute care settings, nurses with autonomy can advocate for their patients, make critical decisions in time-sensitive situations, and adapt their care plans to meet changing patient needs [34]. By utilizing their nursing agency, nurses can ensure that their care is patient-centered, responsive to individual preferences, and aligned with evidence-based practices [32–34]. The relationship between the study variables and Orem’s general theory of nursing is illustrated in Fig. 1.

In Saudi Arabia, most healthcare sectors have complained of a high nurse turnover rate in the last few years [30, 31, 35]. Conducting a study on the autonomy of nurses in acute care settings is crucial in tackling healthcare organizations’ challenges, including nurse turnover, burnout, and low job satisfaction [30, 35, 36], which ultimately impacts patient outcomes. Not only does this research bridge a gap in the nursing literature, but it also adds valuable insights to the nursing field. By identifying the factors that influence the level of autonomy among Saudi staff nurses in acute care settings, this study can inform policies and practices related to nursing autonomy in the country. Moreover, it can augment nursing education and provide healthcare organizations with strategies to support staff nurses in making autonomous decisions, ultimately improving their work environments and overall well-being. Therefore, this study aimed to assess Saudi nurses’ autonomy level in acute care settings and examine the influence of demographic factors on Saudi nurses’ autonomy level in acute care settings in Saudi Arabia.

The study’s identification of demographic and clinical factors influencing autonomy levels can also help guide nursing education and training programs in Saudi Arabia and beyond, ensuring that nurses are better equipped to make independent decisions regarding patient care and unit operations. The study findings could also be utilized for upcoming research to comprehend better the multifaceted factors that impact Saudi nurses’ acceptance of independent decision-making in their workplaces. Moreover, this study may enhance nurses’ perceptions of their work environments and inform efforts to improve nursing practice.

## Methods

### Study design and patients

A cross-sectional correlational design was used to assess Saudi nurses’ autonomy level in critical care settings and examine the influence of demographic factors on the level of autonomy. Descriptive correlational designs are used to learn more about natural events, identify phenomena, and clarify factors associated with those phenomena [37].

### Sampling and setting

This study used a non-probability convenience sampling approach to recruit 212 staff nurses working in critical care settings (Medical ICU, surgical ICU, pediatric ICU, and CCU) from five Saudi governmental hospitals in al jouf region. The current study determined the sample size based on power analysis using G*Power 3.1.9.2 software [38]. The researcher has determined a medium effect size (f = 0.1) based on the current literature review, a significance level of α = 0.05, and a power of 80%. The estimated sample size for a multiple linear regression model with seven variables was 143 nurses. More participants were recruited to have a more representative sample and overcome the expected attrition rate. A total of 317 nurses were invited to fill out the survey. In total, a sample of 212 nurses responded to the invitation, representing approximately a 67% response rate.

This study has defined inclusion and exclusion criteria for its sample population. The inclusion criteria consist of nurses who are currently working full-time in acute care settings and having at least one year of experience in acute care settings within the last three years. However, the exclusion criteria include nurses who have less than one year of experience in acute care settings, nurses who have been working in non-critical care units for the past three years, and nurses who are on leave or vacation during the data collection period.

### Instrument

The researcher utilized two section instrument to address the aim of this study. The first section includes sociodemographic characteristics of nurses (age, gender, marital status, shift worked, level of education, length of experience in nursing, and length of experience in the current critical care setting). The second section is the original scale of MA Blegen, C Goode, M Johnson, M Maas, L Chen and S Moorhead [39], which consists of 42 items measured on an ordinal level of measurement (Likert scale) ranging from 1 to 5. The lowest score (1) represents nurses with no authority or accountability in their clinical setting. Whereas the highest score (5) represents nurses with full authority or accountability. The Likert scale also includes intermediate points, such as nurses assuming authority and accountability when asked (2), sharing authority and accountability with others (3), and consulting with others and participating in group decisions (4).

Belgen’s instrument has two primary subscales, each consisting of 21 items. The first subscale contains items that discuss patient care decisions. The second subscale contains items that discuss unit operation decisions. The original Belgen scale’s psychometric properties were found to be satisfactory and reported by MA Blegen, C Goode, M Johnson, M Maas, L Chen and S Moorhead [39]. The subscales’ Cronbach alpha values ranged from 0.78 (patient care decisions) to 0.92 (unit operation decisions). Previous studies [40, 41] have confirmed that the scale’s content validity was adequate. The scale is designed to identify areas where nurses may feel they lack autonomy in their work and can be used to inform interventions aimed at improving nurses’ autonomy and job satisfaction. The reliability coefficient (Cronbach’s alpha) of the subscales in this study was 0.96 and 0.94 for the patient care and unit operations decisions subscales, respectively, indicating good internal consistency.

### Data collection

To ensure maximum participation, the study utilized multiple methods of data collection. In addition to the online survey questionnaire created with Google Forms, a few printed copies of the questionnaire were also made available to participants. The printed surveys included the same questions as the online version and were posted on the notice boards of the nursing service department of the five government hospitals. Instructions on how to complete and return the printed surveys were included with the notification. Participants were encouraged to return the completed printed surveys to designated collection boxes located in the nursing service department or to scan the QR code on the notice board to access the online survey. The web link invitation to participate in the study was forwarded to staff nurses via email and various social media platforms such as Messenger, Instagram, and WhatsApp to enhance participation further. Informed consent was obtained from all participants at the beginning of the survey, and they were informed about the purpose of the study, its benefits and risks, and why it was being conducted. The data collection process took place over three months from November 2022 to January 2023. The study ensured the confidentiality and anonymity of participants by storing the collected data in a password-protected account in Google Drive. During the data collection process, nurses took an average of 25 to 30 min to complete both the online and printed survey questionnaire.

### Statistical analysis

The study variables were coded and analyzed using SPSS Statistics version 25. Descriptive statistics, such as means and standard deviations, were used to describe the demographic information, and nurses’ autonomy scale items, while categorical variables were presented as percentages and frequencies. To determine the average score for each subscale (patient care and unit operations), the scores of all items within each subscale are added together, and then divided by the total number of items in that particular subscale. Furthermore, multiple linear regression was conducted to identify the predictors of work autonomy among nurses, taking into account sociodemographic and clinical characteristics.

### Ethical consideration

This study was approved by the bioethics institutional review board of Jouf University, in accordance with the Helsinki declaration [42]. The study received the approval number (4-02-44). To ensure confidentiality and anonymity, several strategies were implemented, such as assigning a unique code number to each nurse and reporting all data in aggregate form. The data was handled electronically through a password-protected account, and participants provided electronic informed consent before completing the survey on a Google form. The electronic form of obtaining informed consent was approved by the bioethics institutional review board of Jouf University (Approval no. 4-02-44).

## Results

### Nurses’ sociodemographic and clinical characteristics

The survey was completed by 212 nurses working in critical care units. Most of the sample was female (60.8%), while 39.2% were male. Regarding marital status, most nurses were married, covering 73.1% of the sample, followed by (single and divorced) nurses at 21.2% and 5.7%, respectively. Regarding work shifts, most nurses worked in the morning, while a minority of 22.2% worked in a shift rotation. In addition, almost all nurses worked full-time, with only 1.4% working part-time. The highest level of education held by most nurses was a Bachelor’s degree, with 48.1% of the sample having attained this level of education. This was followed by nurses who held a Master’s degree (25.9%), while 24.5% had a diploma, and only 1.4% held a doctorate. In terms of age, most nurses were between 25 and 34 years old, accounting for 52.4% of the sample. Those aged 35 years or more accounted for 40.5%, while a small percentage of nurses were either younger than 25 years old at 7.1%.

Regarding nursing experience, most nurses had more than ten years of experience, representing 59.9% of the sample. Those with 5–9 years of experience accounted for 15.6%, while a smaller percentage of nurses had less than one year, one to two years, or three to four years of experience, at 7.1%, 7.5%, and 9.9%, respectively. In terms of experience in their current area of employment, most nurses had less than one year of experience (12.3%). In contrast, 13.2% had one to two years of experience, and 20.8% had three to four years of experience. A relatively smaller percentage of nurses had 5–9 years of experience at 17.9%, while more than ten years of experience in their current area of employment was reported by 35.8% of nurses. Details of the sociodemographic and clinical characteristics are illustrated in Table 1.

**Table 1:** Descriptive statistics (frequencies and percentages) of nurses’ sociodemographic and clinical characteristics (N = 212)

Variables	N	%
Gender		
Male	83	39.2%
Female	129	60.8%
Marital Status		
Single	45	21.2%
Married	155	73.1%
Divorced	12	5.7%
Shift Worked		
Morning	165	77.8%
Shift Rotation	47	22.2%
Time Commitment for Work		
Full Time	209	98.6%
Part Time	3	1.4%
Highest Level of Education		
Diploma	52	24.5%
BSc	102	48.1%
Master	55	25.9%
Doctorate	3	1.4%
Age		
< 25 years	15	7.1%
25–34	111	52.4%
≥ 35 years	86	40.5%
Years of Nursing Experience		
< 1 year	15	7.1%
1–2 years	16	7.5%
3–4 years	21	9.9%
5–9 years	33	15.6%
≥ 10 years	127	59.9%
Years of Experience in Current Area of Employment		
< 1 year	26	12.3%
1–2 years	28	13.2%
3–4 years	44	20.8%
5–9 years	38	17.9%
≥ 10 years	76	35.8%

### Nurses’ work autonomy in making patient care decisions

Table 2 displays the mean and standard deviation for 21 patient care decision tasks, with higher means indicating greater levels of independent authority and accountability for nurses on each task. The task with the highest mean is “Prevent patient falls” with a mean of 3.84 and a standard deviation of 1.33, indicating that nurses have a relatively high level of authority and accountability in preventing falls among patients. The next highest mean is for “Prevent skin breakdown” with a mean of 3.69 and a standard deviation of 1.26, suggesting that nurses have a significant level of independent authority and accountability for this task. “Teach healthcare promotion activities” has a mean of 3.62 and a standard deviation of 1.31, indicating that nurses also have a high level of independent authority and accountability for promoting health among patients. The mean score value for “Teach self-care activities” is 3.56, which suggests that staff nurses have a moderate level of authority and accountability in teaching patients to care for themselves. “Question doctor order” has a mean score of 3.55, indicating that staff nurses have moderate independent authority and accountability for questioning physician orders. Other tasks with a mean greater than 3.0 include “Consult with a medical doctor and other professionals” (M = 3.51), “Serve as a patient advocate” (M = 3.51), “Decide time to administer care” (M = 3.39), and “Decide for pain management” (M = 3.26).

In contrast, the task with the lowest mean is “Order diagnostic test” with a mean of 2.27, suggesting that staff nurses have a relatively low level of authority and accountability for ordering diagnostic tests. The second lowest mean is for “Determine the day of discharge” with a mean of 2.61, indicating that staff nurses also have a relatively low level of authority and accountability for deciding when patients can be discharged from the hospital. The mean score of the total subscale of staff nurses’ work autonomy in patient care decisions is 3.25. This suggests that, on average, staff nurses had moderate autonomy in making patient care decisions, with a relatively wide range of scores among staff nurses.

**Table 2:** Descriptive statistics (mean and standard deviation) of nurses’ work autonomy level in making patient care decisions (N = 212)

Patient care decision tasks	Min	Max	95% CI	M	SD
1. Take measures to avoid patients falling	1	5	(3.66–4.02)	3.84	1.33
2. Prevent the development of skin ulcers	1	5	(3.52–3.86)	3.69	1.26
3. Educate patients on healthcare promotion activities	1	5	(3.45–3.80)	3.62	1.31
4. Teach patients self-care tasks	1	5	(3.37–3.74)	3.56	1.34
5. Teach about patient medication	1	5	(3.39–3.73)	3.56	1.28
6. Clarify physician’s orders by asking questions	1	5	(3.36–3.73)	3.55	1.38
7. Consult with medical professionals and other experts	1	5	(3.32–3.69)	3.51	1.37
8. Act as an advocate for the patient	1	5	(3.32–3.70)	3.51	1.39
9. Determine the best time to administer care	1	5	(3.20–3.57)	3.39	1.38
10. Collaborate with patients to plan their care	1	5	(3.06–3.43)	3.25	1.39
11. Make decisions about pain management	1	5	(3.08–3.45)	3.26	1.37
12. Handle complaints from physicians	1	5	(2.90–3.30)	3.10	1.48
13. Refuse to carry out orders from physicians, if necessary	1	5	(2.97–3.36)	3.17	1.41
14. Inform patients about the risks associated with surgery	1	5	(2.95–3.34)	3.15	1.44
15. Create educational materials for patients	1	5	(2.88–3.25)	3.06	1.36
16. Refer patients to other healthcare professionals	1	5	(2.63–3.01)	2.82	1.41
17. Address complaints from individual patients	1	5	(2.93–3.32)	3.13	1.44
18. Follow orders for “as needed” medications	1	5	(2.93–3.34)	3.13	1.50
19. Determine the appropriate day for discharge	1	5	(2.41–2.81)	2.61	1.46
20. Discuss alternatives with physicians	1	5	(2.84–3.25)	3.04	1.51
21. Order diagnostic tests as needed	1	5	(2.07–2.47)	2.27	1.48

Min, minimum score; Max, maximum score; CI, the 95% confidence interval for the mean; M, Mean, SD, standard deviation

### Nurses’ work autonomy in making unit operations decisions

Table 3 displays descriptive statistics for various tasks related to nurses’ work autonomy in making unit operation decisions. In general, the staff nurses in this study had moderate autonomy in making unit operation decisions. The tasks with the highest mean values for nurses were “develop unit goals” (M = 3.42), “determine meal times and personal breaks " (M = 3.29), and “implement new ideas, i.e., class” (M = 3.24). This suggests that staff nurses have more autonomy in these areas and can make decisions independently. In contrast, the tasks with the lowest mean values for staff nurses were “plan the unit’s annual budget” (M = 2.22), “identify reasons for variances in the unit budget” (M = 2.27), and “define the job description of staff nurses” (M = 2.56). This indicates that staff nurses have less autonomy in these areas and may not have as much independent decision-making. The mean score of the total subscale of staff nurses’ work autonomy in unit operation decisions is 2.91. This suggests that, on average, staff nurses have a moderate level of autonomy in making decisions related to unit operations.

**Table 3:** Descriptive statistics (mean and standard deviation) of nurses’ work autonomy level in making unit operation decisions (N = 212)

Unit operation decision tasks	Min	Max	95% CI	M	SD
1. Develop unit goals	1	5	(3.23–3.60)	3.42	1.37
2. Determine personal breaks and meal times	1	5	(3.10–3.48)	3.29	1.42
3. Implement new ideas, i.e., class	1	5	(3.05–3.45)	3.24	1.40
4. Assign patients to staff members	1	5	(3.03–3.41)	3.22	1.40
5. Update and modify standards for care	1	5	(2.97–3.35)	3.16	1.38
6. Set agendas for staff meetings	1	5	(2.92–3.31)	3.11	1.43
7. Organize trading hours for the unit	1	5	(2.98–3.36)	3.17	1.43
8. Decide on the method of delivering care	1	5	(2.88–3.25)	3.07	1.38
9. Revise unit procedures within the department	1	5	(2.80–3.21)	3.00	1.48
10. Update and modify unit policies	1	5	(2.80–3.20)	3.00	1.46
11. Participate in department/hospital committees	1	5	(2.85–2.24)	3.04	1.45
12. Determine quality assurance indicators	1	5	(2.79–3.19)	2.99	1.47
13. Schedule individual work hours	1	5	(2.77–3.16)	2.97	1.43
14. Initiate research activities	1	5	(2.77–3.17)	2.97	1.48
15. Conduct peer review evaluations for nurses	1	5	(2.68–3.03)	2.85	1.30
16. Select new equipment and supplies	1	5	(2.54–2.90)	2.72	1.32
17. Define the job description of staff nurses	1	5	(2.38–2.74)	2.56	1.34
18. Present unit in service	1	5	(2.24–2.66)	2.45	1.53
19. Identify reasons for variances in unit budget	1	5	(2.09–2.44)	2.27	1.29
20. Plan the unit’s annual budget	1	5	(2.04–2.41)	2.22	1.38
21. Conduct interviews and choose new staff members	1	5	(2.21–2.62)	2.41	1.51

Min, minimum score; Max, maximum score; CI, the 95% confidence interval for the mean; M, Mean, SD, standard deviation

### Overall saudi Nurses’ work autonomy

The mean score for total autonomy for the entire sample was 3.08, with patient care decisions autonomy at 3.25 and unit operations decisions autonomy at 2.91. These findings suggest that nurses perceived their overall work autonomy as moderate and felt more independent in making patient care decisions than in making decisions related to unit operations.

### Predictors of nurses’ work autonomy

The overall regression model was statistically significant (R^2^ = .32, F (16, 195) = 5.87, p < .001) and explains approximately 32% of nurses’ work autonomy variance. Among the independent variables, the dummy coded variable of “Master/doctorate” was found to have a statistically significant positive relationship with nurses’ work autonomy (β = .19, t (195) = 2.10, p = .037). This suggests that nurses who have a master’s or doctoral degree have higher levels of work autonomy compared to those with lower levels of education. In addition, the dummy coded variables “less than one year of experience in Current Area of Employment” (β = − .29, t (195) = − .90, p = .004) and “1–2 years of experience in Current Area of Employment” (β = − .18, t (195) = -2.34, p = .020) were also found to have a statistically significant negative relationship with nurses’ work autonomy. This suggests that nurses with less experience in critical care settings have less work autonomy than those with more experience in critical care units. The remaining variables, including Age, gender, marital status, shift worked, and years of nursing experience were not found to be significantly related to nurses’ work autonomy. Table 4 illustrates the results of the standard multiple linear regression model.

**Table 4:** **Standard multiple linear regression that predicts nurses work autonomy from the sociodemographic and clinical characteristics** (**N = 212)**

Variable	B	SE	CI	β	t	p
Gender	-0.02	0.14	[-0.31, 0.26]	-0.01	-0.17	0.863
Shift worked	0.33	0.19	[-0.04, 0.70]	0.14	1.75	0.081
Marital Status						
D1 (Single)	-0.10	0.21	[-0.51, 0.32]	-0.04	-0.46	0.644
D2 (Divorced)	0.12	0.29	[-0.45, 0.68]	0.03	0.40	0.688
Reference group (Married)						
Level of education						
D1 (Diploma)	0.36	0.25	[-0.12, 0.85]	0.16	1.47	0.144
D2 (Master/doctorate)	0.43	0.21	[0.03, 0.84]	0.19	2.10	0.037
Reference group (Bachelor)						
Age						
D1 (less than 25 years)	-0.18	0.76	[-1.68, 1.31]	-0.05	-0.24	0.809
D2 (25–34 years)	-0.34	0.18	[-0.71, 0.02]	-0.17	-1.88	0.061
Reference group (More than 35 years)						
Years of Nursing Experience						
D1 (< 1 year)	-0.63	0.63	[-1.87, 0.61]	-0.17	-1.00	0.317
D2 (1 to 2 years)	-0.44	0.34	[-1.10, 0.22]	-0.12	-1.32	0.189
D3 (3 to 4 years)	-0.04	0.35	[-0.72, 0.65]	-0.01	-0.11	0.915
D4 (5 to 9 years)	0.06	0.28	[-0.50, 0.62]	0.02	0.21	0.830
Reference group (≥ 10 years)						
Years of Experience in Current Area of Employment						
D1 (< 1 year)	-0.88	0.30	[-1.48, -0.28]	-0.29	-2.90	0.004
D2 (1–2 years)	-0.53	0.23	[-0.97, -0.08]	-0.18	-2.34	0.020
D3 (3–4 years)	-0.42	0.23	[-0.88, 0.03]	-0.17	-1.84	0.068
D4 (5–9 years)	-0.21	0.21	[-0.62, 0.20]	-0.08	-1.02	0.308
Reference group (≥ 10 years)						
Overall model F (16,195) = 5.87, p < .001, R2 = 0.32						

b, unadjusted regression coefficient; β, adjusted regression coefficient; SE, the standard error of the coefficient; CI, the 95% confidence interval for the coefficient; t, the t-value for the coefficient; and D, dummy variable

## Discussion

Professional autonomy is a critical aspect of nursing practice that allows nurses to make independent decisions about patient care and unit operations [1, 8–10, 15, 19, 43–46]. The present study investigated the predictors and levels of professional autonomy among critical care nurses in Saudi Arabia. The current study found that the overall mean of the autonomy scale was 3.08, which is higher than the midpoint, indicating a moderate level of professional autonomy. The findings are consistent with several local and international studies, such as [4], [1], [43], [45], [46], [30], [41], and [8]. This moderate autonomy level could be related to the frequent and rapid changes in the healthcare system in the country, which limits nurses’ control over patient care and the unit’s daily operations [24, 36, 44].

According to the staff nurses’ reports, they felt a greater sense of autonomy while making decisions about patient care than when making decisions about unit operations. This finding is consistent and supported by previous research [8, 41, 43]. For instance, a recent study conducted among hospital staff nurses by LJ Labrague, DM McEnroe-Petitte and K Tsaras [8] found that nurses in hospital care settings reported having a greater sense of autonomy in making patient care decisions than decision-making about unit operations which may be because patient care is the primary focus of nursing practice [8]. In contrast, unit operations decisions like resource allocation, scheduling, and staffing may be more closely tied to administrative policies and protocols. These decisions may be influenced by factors outside the nurse’s control, such as and organizational policies and budget constraints [8, 41, 43].

The findings on the level of autonomy among nurses in acute care settings for various patient care tasks are consistent with other published studies. For instance, a study conducted among 807 staff nurses by ED Papathanassoglou, M Tseroni, A Karydaki, G Vazaiou, J Kassikou and M Lavdaniti [47] found that nurses in acute care settings have a high level of autonomy in areas such as monitoring patients, administering medication, and providing patient education. Similarly, another study by J Hagan and DL Curtis Sr [29] found that nurses in acute care settings have a high level of autonomy in tasks such as developing care plans, assessing patients, and providing patient education. However, the findings that nurses have a relatively low level of authority for determining the day of discharge and ordering diagnostic tests are also consistent with previous studies. For example, a study by KK Iliopoulou and AE While [15] found that nurses in acute care settings have limited involvement in decision-making regarding patient discharges. Another study by ED Papathanassoglou, M Tseroni, A Karydaki, G Vazaiou, J Kassikou and M Lavdaniti [47] found that nurses in acute care settings often do not order diagnostic tests. These findings can inform discussions around the roles and responsibilities of nurses in healthcare settings, particularly in acute care settings.

Several international studies have identified various factors contributing to determining nurses’ work autonomy, such as years of experience, nursing specialization, education level, and clinical training [1, 2, 8, 19, 41, 48]. This study found that staff nurses with a higher educational level, particularly those with a master’s or doctorate and those with more years of experience in the acute care setting, had a greater sense of autonomy in their jobs. These findings are consistent with previous research showing a significant positive correlation between nurses’ level of education and professional autonomy [2, 8, 41, 48]. The study also highlights the importance of clinical judgment, which is essential for autonomous nursing practice and can only be developed through higher education, extensive clinical experience, and adequate training [1, 19]. These findings suggest that investing in the training and education of nurses can contribute to increasing their professional autonomy and improving the quality of care [1, 19, 41]. Moreover, the study’s results can inform policymakers and nursing administrators in developing strategies that aim to promote nurses’ professional development and increase their autonomy level. For example, hospitals and healthcare organizations can prioritize offering training and educational programs for nurses to enhance their clinical expertise, knowledge, and skills [2, 5, 15, 40, 41].

Organizational culture, professional development opportunities, leadership style, and adequate staffing levels can influence nurses’ autonomy in acute care settings [2, 19, 20, 27, 28, 40, 43, 46]. An environment that values nursing professional autonomy and provides ongoing education and training opportunities promotes greater independence for nurses [2, 19, 20, 27, 28, 40, 43, 46]. The healthcare system in Saudi Arabia has undergone considerable investment, leading to a rise in the number of healthcare facilities and hospitals. This has subsequently improved the work environment for nurses [49, 50].

Professional autonomy is closely related to empowerment and is a critical aspect of nursing practice [1, 2, 5, 40, 41]. According to current literature, empowering nurses leads to better patient outcomes, higher job satisfaction, and decreased burnout rates [2, 5, 40, 41, 51, 52]. Moreover, nurse empowerment has been linked to better organizational performance, increased trust in leadership, and improved collaboration among healthcare providers [1, 2, 5, 40, 41]. Nurses’ empowerment and autonomy should be fostered from the undergraduate level to enable them to become independent and critical thinkers in their practice [2, 5, 41]. Professional development of nursing students should include training in clinical decision-making, ethical reasoning, and leadership skills, which can contribute to their professional autonomy.

The current study on professional autonomy among critical care nurses in Saudi Arabia shares several similarities with previous studies [5, 8, 15, 41, 43], which reported similar findings. These studies revealed that nurses feel more independent (autonomous) in making decisions about patient care compared to unit operations. This consistency suggests that prioritizing patient care decision-making aligns with the primary focus of nursing practice across different contexts. Furthermore, the present study’s identification of factors such as education level and years of experience as contributors to nurses’ work autonomy is in line with previous research [8, 15, 19, 34, 41, 43]. These similarities reinforce the existing knowledge on the determinants of professional autonomy and support the generalizability of the findings to different populations and healthcare settings. Despite the similarities, some differences emerge when comparing the current study to previous research. For example, several studies [8, 43–45, 51, 53] focused on hospital care settings, while the present study specifically examined Acute care setting nurses in Saudi Arabia. This distinction suggests that the level of autonomy and the factors influencing it may vary across different nursing specialties and geographic locations.

The study has certain limitations that need to be addressed for future research. Given the nature and dynamics of the autonomy construct being examined, it is recommended that a more comprehensive research design, such as a longitudinal design, be used to monitor changes in the autonomy of nurses over time. Moreover, larger sample sizes, including staff nurses from other provinces, may provide a more representative sample. Additionally, the use of self-report scales may have limited the respondents’ responses. Using mixed-methods or qualitative study design may allow staff nurses to express their perceptions more effectively, which self-report tools may not have captured.

### Implication to practice

The current study has vital implications for clinical practice, emphasizing the need to strengthen staff nurses’ autonomy in unit operations decisions to improve their job satisfaction and overall well-being. Managers and nursing administrators should endeavor to empower staff nurses to make independent decisions related to unit operations to increase their sense of control and job satisfaction. Furthermore, the study’s findings indicate that nurses with less than one year and 1–2 years of experience in acute care settings have less work autonomy than those with more experience. Therefore, nursing managers should be aware of the influence of nurses’ experience on their autonomy and offer support and training to novice nurses to increase their sense of control and confidence in decision-making. In terms of nursing education, the study’s findings indicate that having a higher educational degree positively impacts nurses’ professional autonomy. As a result, nursing education programs should consider incorporating courses or training that improve nurses’ critical thinking and decision-making skills. These abilities are required for nurses to make autonomous decisions about patient care and unit operations, ultimately increasing their professional autonomy.

In the context of nursing law, it is essential to consider the cultural norms and values of Saudi Arabia when formulating regulations that promote professional autonomy for critical care nurses. Cultural norms influence healthcare decision-making, communication styles, and patient preferences. Nursing laws should take into account the cultural expectations and values of Saudi Arabian society to ensure that nursing practice respects and aligns with these norms. This can involve providing guidelines and frameworks considering the cultural context and supporting culturally sensitive, patient-centered care.

## Conclusion

This study aimed to investigate the levels and predictors of professional autonomy among Saudi nurses working in acute care settings. The results showed that nurses have a moderate level of autonomy, with greater autonomy in making patient care decisions than unit operations decisions. Education level, years of experience, and clinical training were significant predictors of nurses’ autonomy, with nurses with higher education and experience reporting greater autonomy. The findings suggest that investing in nurses’ education and training could increase their professional autonomy and improve the quality of patient care. The study’s results can inform policymakers and nursing administrators in developing strategies to promote nurses’ professional development and autonomy.

## Data Availability

The datasets generated and/or analyzed during the current study are not publicly available due to funded ongoing project policy, as the data may be subject to restrictions on data sharing imposed by the funding agency. However, the data are available from the corresponding author on reasonable request.
